# β5i Subunit Deficiency of the Immunoproteasome Leads to Reduced Th2 Response in OVA Induced Acute Asthma

**DOI:** 10.1371/journal.pone.0060565

**Published:** 2013-04-04

**Authors:** Anton Volkov, Stefanie Hagner, Stephan Löser, Safa Alnahas, Hartmann Raifer, Anne Hellhund, Holger Garn, Ulrich Steinhoff

**Affiliations:** 1 Institute for Medical Microbiology and Hygiene, Philipps University of Marburg, Marburg, Germany; 2 Institute for Laboratory Medicine and Pathobiochemistry, Philipps University of Marburg, Marburg, Germany; French National Centre for Scientific Research, France

## Abstract

The immunoproteasome subunit β5i has been shown to play an important role in Th1/Th17 driven models of colitis and arthritis. However, the function of β5i in Th2 dependent diseases remains enigmatic. To study the role of β5i in Th2-driven pathology, β5i knockout (KO) and control mice were tested in different models of experimental allergic asthma. β5i-deficient mice showed reduced OVA/Alum- and subcutaneous/OVA-induced acute asthma with decreased eosinophilia in the bronchoalveolar lavage (BAL), low OVA-specific IgG1 and reduced local and systemic Th2 cytokines. While Th2 cells in the lungs were reduced, Tregs and Th1 cells were not affected. Attenuated asthma in β5i KO mice could not be attributed to defects in OVA uptake or maturation of dendritic cells in the lung. Surprisingly, β5i deficient mice developed HDM asthma which was comparable to control mice. Here, we present novel evidence for the requirement of the β5i immunosubunit to generate a strong Th2 response during OVA- but not HDM-induced acute asthma. The unexpected role of β5i in OVA asthma remains to be clarified.

## Introduction

Allergic asthma is a type I hypersensitivity reaction of the upper and lower respiratory tract which affects more than 300 million people worldwide and therefore, represents one of the most common diseases. It is characterized by recurrent episodes of wheezing, breathlessness, chest tightness and coughing [1]. These manifestations are caused by airway hyper-responsiveness, mucus hypersecretion, lung eosinophilia and accumulation of Th2 cells [2], [3]. Data suggest that in humans, inflammatory reactions of severe asthma are driven by NF-κB [4]. Similarly, in the rat model of OVA-induced allergic asthma, inhibition of NF-κB activation reduced airway eosinophilia and secretion of IL-1α, eotaxin, TNF-α, IL-4, IL-5 and IL-13 [5]. Processing and degradation of many proteins involved in signaling including NF-κB are mediated by proteasomes. The mammalian proteasome is a multicatalytic protease complex which is involved in protein degradation and processing of MHC I class antigens [6]. The 20S proteasome contains three different catalytic activities: caspase-like (β1 subunits), trypsin-like (β2 subunits) and chymotrypsin-like (β5 subunits) [7]. Stimulation with IFN-γ or IFN-α induces replacement of constitutive catalytic subunits by the corresponding immunosubunits, β1i, β2i and β5i leading to the formation of immunoproteasomes [8]. Immunoproteasomes show altered cleavage site specificity with improved MHC-I antigen presentation [9], [10], [11] and increased activation of NF-κB [12], [13].

It has been shown that partial proteasome inhibition by bortezomib or β5i subunit deletion markedly attenuates DSS induced colitis [14], [15] and selective inhibition of β5i by the inhibitor PR-957 ameliorates development of collagen-induced arthritis and decreases pro-inflammatory IL-6, IL-23 and TNF-α cytokines production by peripheral blood mononuclear cells [16]. Surprisingly, the knowledge on proteasome involvement in Th2-driven diseases is scarce. Application of non-specific proteasome inhibitors PS-519 [17] or bortezomib [18] in OVA-induced acute asthma models significantly reduces airway eosinophilia. Isolated eosinophils pretreated with proteasome inhibitor MG-132 show reduced expression of integrin beta-2 (CD18) or stop secreting IL-8 and initiate apoptosis in response to TNF-α [19].

We thus wondered whether specific inhibition of the β5i immunosubunit is an effective strategy for the treatment of acute asthma. Here, we present novel evidence that β5i immunosubunit deletion abrogates OVA-induced acute asthma, as shown by reduced eosinophilia and Th2 responses. However, lack of the β5i immunosubunit did not show any beneficial effects in the clinically relevant HDM asthma model.

## Results

### Reduction of the asthmatic phenotype in β5i KO mice in experimental OVA/Alum model

**Figure 1:**
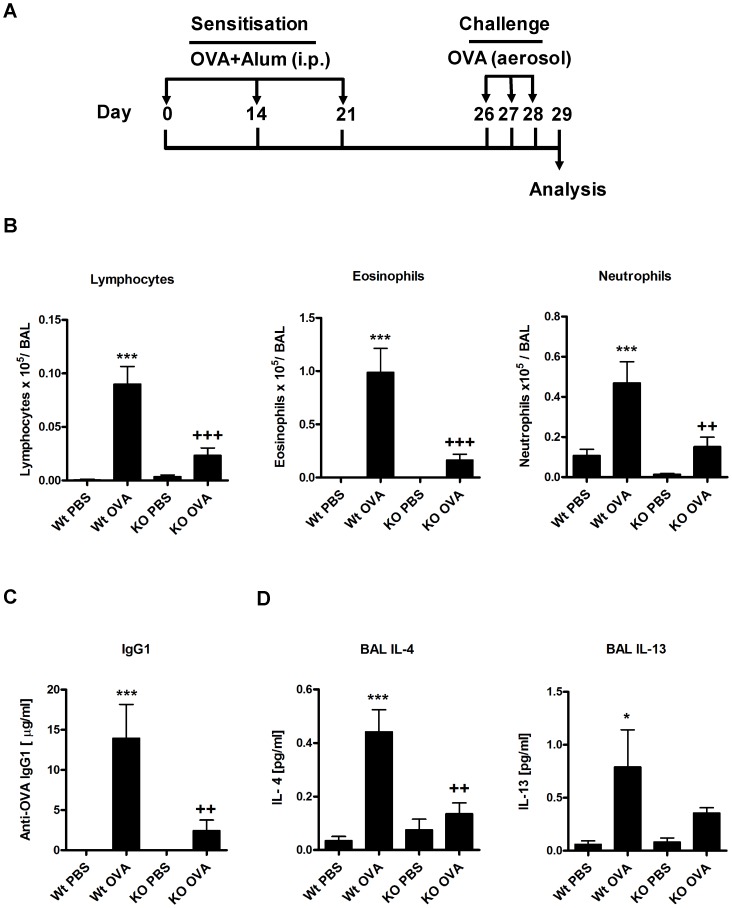
OVA/Alum acute asthma is reduced in β5i KO mice. (A) OVA/Alum asthma induction protocol. (B) Differential BAL cytospins cell counts. (C) OVA-specific antibody titers in serum. (D) BAL Th2 cytokines concentrations. The data are representative of three independent experiments and shown as the mean ± SEM (n = 6–10). (*) and (+) represent comparison of PBS vs OVA and Wt OVA vs KO OVA treatment groups respectively. *^/+^P<0.05, **^/++^P<0.01, ***^/+++^P<0.001.

**Figure 2:**
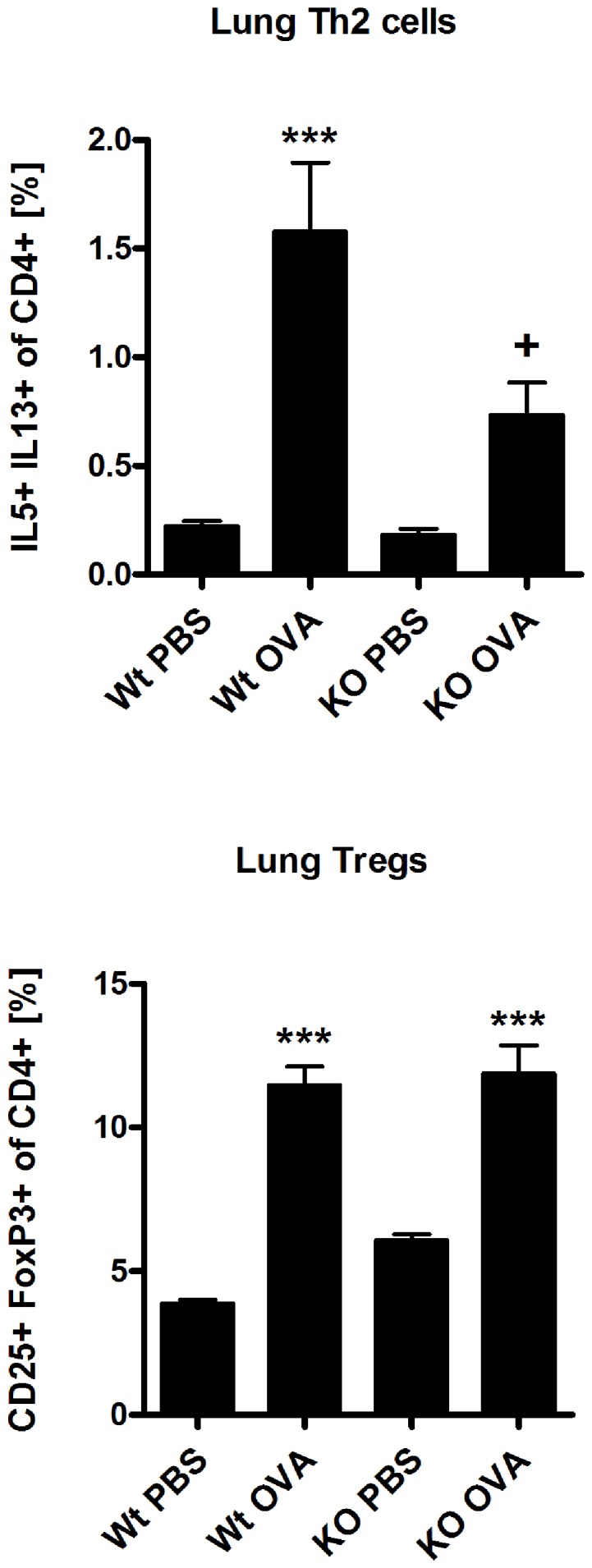
Lung Th2 and Treg cell profile of Wt and β5i KO mice during OVA/Alum acute asthma. The percentages of IL5+ IL13+ of CD4+ and CD25+ FoxP3+ of CD4+ T cells were measured by flow cytometry of homogenized lungs. The data are representative of two independent experiments and shown are the mean ± SEM (n = 6). (*) and (+) represent comparison between PBS vs OVA and Wt OVA vs KO OVA treatment groups, respectively. *^/+^P<0.05, ***^/+++^P<0.001.

**Figure 3:**
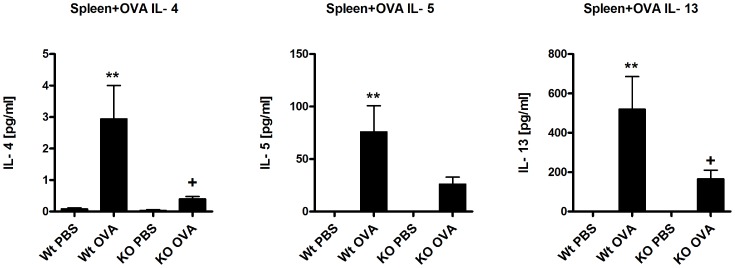
Reduced Th2 response in OVA reactivated splenocytes. The measurement of IL-4, IL-5 and IL-13 cytokines was performed by cytometric bead array (BD) from the supernatants of OVA reactivated splenocytes after 72 h of reactivation. The data are representative of two independent experiments and shown as the mean ± SEM (n = 6). (*) and (+) represent comparison of PBS vs OVA and Wt OVA vs KO OVA treatment groups respectively. *^/+^P<0.05, **^/++^P<0.01.

To investigate the role of β5i subunit of the immunoproteasome, C57BL/6N (Wt) and β5i KO mice were sensitized and challenged either with PBS/OVA or OVA/OVA as shown in Figure 1A. BAL fluid was collected and differential cell counting was performed as described in the materials and methods section. In comparison to Wt mice, β5i KO mice demonstrated reduced recruitment of inflammatory cells as shown by the lower number of eosinophils, lymphocytes and neutrophils in the BAL (Figure 1B). Further, OVA specific IgG1 titers (Figure1C) and the Th2 cytokines IL-4 and IL-13 produced by BALs were significantly decreased in β5i KO mice as compared to Wt controls (Figure 1D). Additionally, lung Th2 and Treg cells profile of the Wt and β5i KO mice was assessed by flow cytometry from the homogenized lungs. We found that β5i KO mice had decreased Th2 cells percentage (0.8±0.35% vs 0.4±0.05%, +P<0.05) in comparison to the Wt during the asthma (Figure 2, Figure S1). Despite increased percentage of lung Tregs during asthma, no difference between the Wt and β5i KO was demonstrated (Figure 2, Figure S2). Similarly, no significant differences in Th1 lung cells content was observed, although a slight decrease in Th1 cells in the Wt asthma group was detectable (data not shown). To further investigate whether the observed reduction of pulmonary Th2 responses in β5i KO mice is systemic or restricted to the lung, splenocytes from OVA-asthma induced Wt and β5i KO mice were *in-vitro* restimulated with OVA and cytokines were measured in the culture supernatants. Unexpectedly, splenocytes of OVA sensitized and challenged β5i KO mice produced less IL-4, IL-5 and IL-13 as compared to Wt mice (Figure 3).

### Effects of β5i KO during OVA acute asthma are independent of Alum

**Figure 4:**
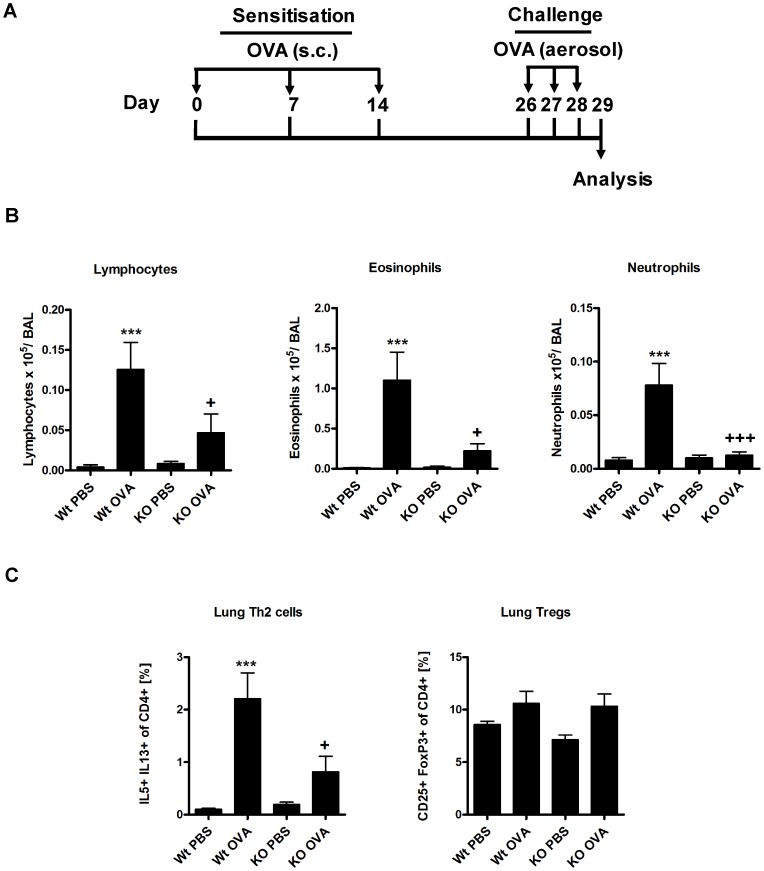
Subcutaneously OVA induced acute asthma is reduced in β5i KO mice. (A) OVA subcutaneous asthma induction protocol. (B) Differential BAL cytospins cell counts. (C) Lung Th2 and Treg cells profile. The percentages of IL5+ IL13+ and CD25+ FoxP3+ of CD4+ T cells were measured by flow cytometry from the homogenized lungs. The data are representative of two independent experiments and shown as the mean ± SEM (n = 6–8). (*) and (+) represent comparison of PBS vs OVA and Wt OVA vs KO OVA treatment groups respectively. *^/+^P<0.05, ***^/+++^P<0.001.

It is known that Alum affects dendritic cell activation and consequent antigen presentation [20]. To exclude any influence of alum on the asthmatic phenotype, we employed our well-established, Alum-free subcutaneous OVA-induced asthma model (Figure 4A) [21]. Similarly to OVA/Alum asthma, β5i KO mice demonstrated a significant reduction in the allergic inflammation as compared to Wt mice. The frequency of eosinophils, lymphocytes and neutrophils in the BAL was significantly diminished in the mutant mice (Figure 4B). Analysis of lung T-cells revealed comparable reduction of Th2 cells in β5i KO mice (Figure 4C), while Tregs were equally increased in both the Wt- and β5i KO mice (Figure 4C), which is comparable to the OVA/Alum asthma (Figure 2).

### Reduced FITC-OVA uptake by lung inflammatory dendritic cells (iDCs) in β5i KO

**Figure 5:**
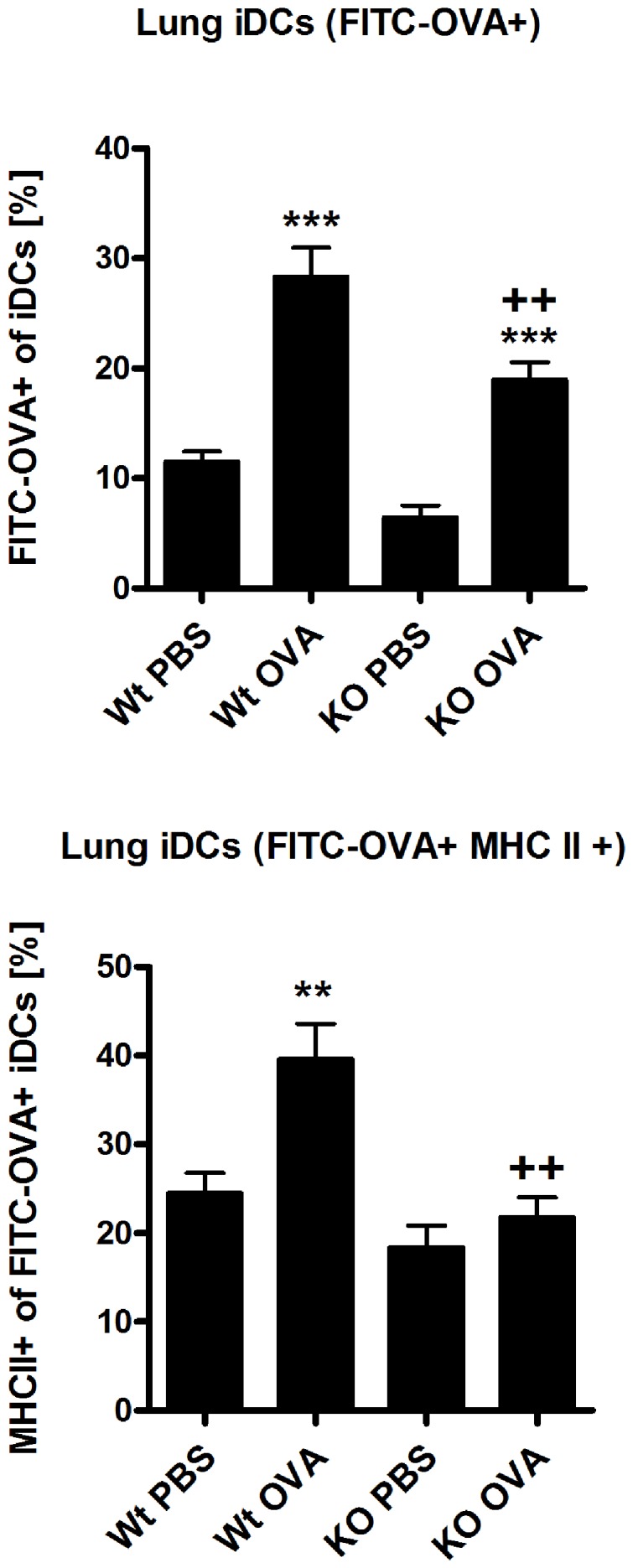
Lung inflammatory dendritic cells (iDCs) show reduced FITC-OVA uptake and maturation in β5i KO mice. The percentages of FITC−OVA+ of iDCs and MHC II+ of FITC-OVA iDCs were measured by flow cytometry from the homogenized lungs 24 h post intranasally FITC-OVA application in mice that have been sensitized with OVA/Alum. Data are representative of two independent experiments and shown as the mean ± SEM (n = 10). (*) and (+) represent comparison of PBS vs OVA and Wt OVA vs KO OVA treatment groups respectively. **^/++^P<0.01, ***^/+++^P<0.001.

The β5i subunit of the immunoproteasome plays an important role in NF-κB activation [12], [13] and thus promotes maturation of dendritic cells [22]. To investigate whether reduced Th2 response during OVA/Alum asthma in β5i KO mice is due to impairment in DC maturation, mice were challenged intranasally with a FITC-OVA conjugate and FITC-OVA positive iDCs (F4/80−CD11c+CD11b+Ly6C+B220−, gating strategy in Figure S3) were analyzed on the following day. Compared to WT mice, iDCs from β5i KO mice revealed significantly decreased FITC-OVA uptake and reduced maturation as shown by low expression of MHC II molecules (Figure 5). Similar analysis of conventional lung DCs (cDCs) in β5i KO mice revealed no differences between WT mice (data not shown). Further analysis of the FITC-OVA iDCs migration to the mediastinal lymph nodes showed no difference in the distribution between the Wt and β5i KO mice (data not shown), suggesting that an intrinsic defect of iDCs in β5i KO mice might contribute to the observed phenotype.

### Th2 response is not restored by transfer of normal dendritic cells into β5i KO mice

**Figure 6:**
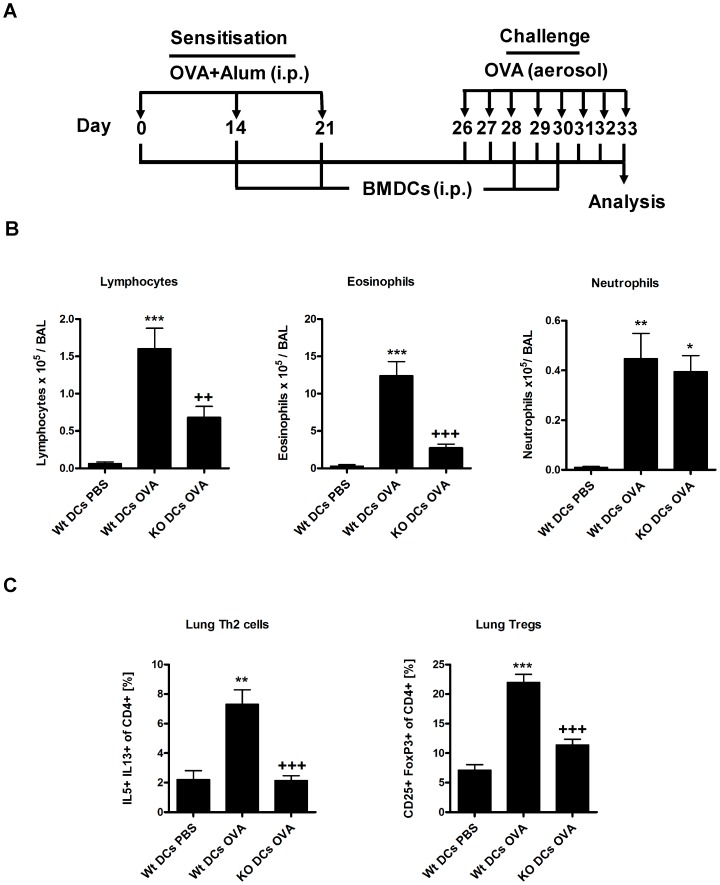
Reduced pulmonary Th2 response is not due to intrinsic defects of iDCs in β5i KO mice. (A) OVA/Alum asthma induction protocol. (B) Differential cell count of BAL cytospins. (C) Lung Th2 and Treg cell profile. Wt bone marrow derived dendritic cells (BMDCs) were injected i.p. with 0.8×10^6^/mouse per injection. The percentages of IL5+ IL13+ and CD25+ FoxP3+ of CD4+ cells were measured by flow cytometry from the homogenized lungs. The data are representative of two independent experiments and shown as the mean ± SEM (n = 6). (*) and (+) represent comparison of PBS vs OVA and Wt OVA vs KO OVA treatment groups respectively. *^/+^P<0.05, **^/++^P<0.01, ***^/+++^P<0.001.

**Figure 7:**
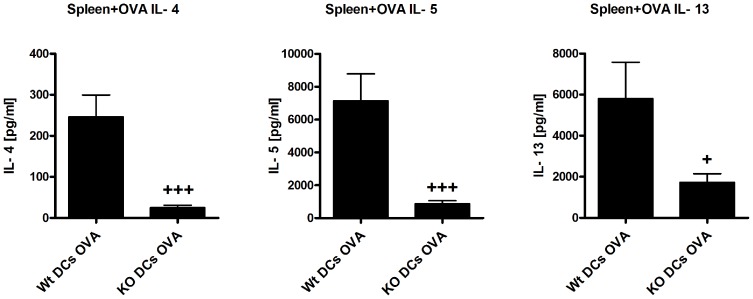
Th2 response is not restored in OVA reactivated splenocytes of BMDCs transferred β5i KO mice. Wt bone marrow derived dendritic cells (0.8×10^6^ BMDCs/mouse) were injected i.p. The cytokines IL-4, IL-5 and IL-13 were measured by cytometric bead array (BD) from supernatants of OVA reactivated splenocytes after 72 h of reactivation. Data are representative of two independent experiments and shown as the mean ± SEM (n = 6–12). (*) and (+) represent comparison of PBS vs OVA and Wt OVA vs KO OVA treatment groups respectively. *^/+^P<0.05, ***^/+++^P<0.001.

To study whether Th2 driven responses can be rescued in β5i KO mice, we attempted to restore iDCs deficiencies of β5i KO mice by adoptive transfers of *in vitro* generated Wt BMDCs (Figure 6A). Although transfer of BMDCs resulted in increased numbers of iDCs in the lung of β5i KO and control mice (Figure S4A), Wt but not β5i KO mice showed increased allergic inflammation upon OVA-challenge (Figure 6B, C). In order to compare the immune reactivity of OVA-asthma mice with or without transfer of DCs, ratios for differential BAL cell counts, Th2 cells and Th2 cytokines produced by OVA reactivated splenocytes (Figure 7) of Wt OVA to KO OVA and Wt DCs OVA to KO DCs OVA groups were calculated. We obtained the following ratios for lymphocytes (5 and 2.3), eosinophils (5 and 4), neutrophils (4 and 1), Th2 cells (2 and 3.5), IL-4 (8 and 10), and IL-13 (3 and 3). In both groups, similar ratios for eosinophils, Th2 cells, and IL-4 and IL-13 suggest that adoptive transfer of BMDCs was not able to complement the Th2 defect in β5i KO mice. Nevertheless, transfer of BMDCs into β5i KO mice increased BAL lymphocytes and completely restored BAL neutrophils numbers to Wt levels (Figure 6B), which corresponds with the increased frequency of lung Th1 cells (Figure S4B).

### Naïve CD4+ T cells from β5i KO mice differentiate into Th2 cells *in vitro*

**Figure 8:**
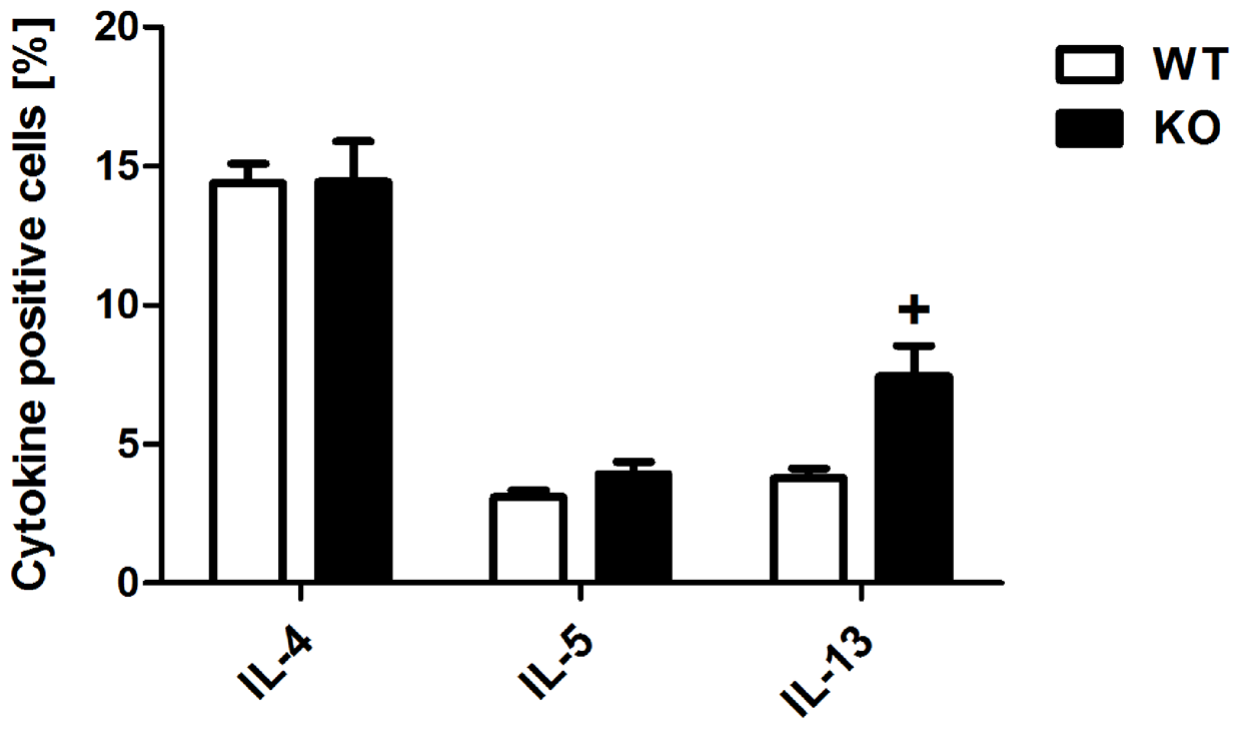
Th2 *in vitro* differentiation is not impaired in naïve β5i KO CD4+ T cells. Total naïve CD4+ cells were isolated by homogenization and subsequent magnetic associated cells sorting (Miltenyi) from spleens and lymphnodes of 6 weeks old mice and differentiated in presence of plate-bound anti-CD3, soluble anti-CD28, anti-IFN-γ and recombinant murine IL-4. The percentages of IL−4+, IL−5+ and IL−13+ of CD4+ T cells were measured by flow cytometry from *in vitro* differentiated Th2 cells. The data are representative of two independent experiments and represented as the mean ± SEM (n = 5). (+) represents comparison of Wt vs KO groups respectively. ^+^P<0.05.

It is well recognized that the β5i subunit of the immunoproteasome plays an important role in Th1 [14], [15] and Th17 [16] responses. However, no information is available regarding its involvement in Th2 cell development. Hence, we analyzed Th2 differentiation of naïve CD4+ T cells from β5i KO mice in order to understand its function in Th2 transcriptional program and cytokine production. Antigen-independent differentiation of β5i KO naïve CD4+ cells revealed that Th2 cells from β5i KO mice produced normal amounts of IL-4, IL-5 and IL-13 (Figure 8, Figure S5), suggesting intact Th2 differentiation.

### Th2 response reduction during acute asthma in β5i KO mice is OVA specific

**Figure 9:**
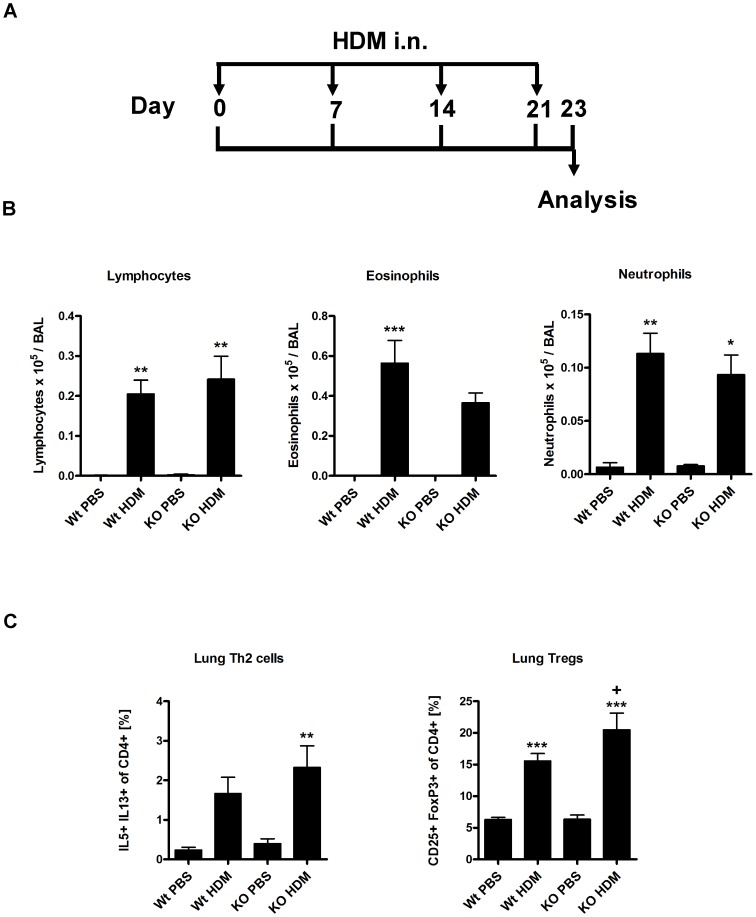
HDM induced acute asthma is not reduced in β5i KO mice. (A) HDM asthma induction protocol. (B) Differential BAL cytospins cell counts. (C) Lung Th2 and Treg cells profile. The percentages of IL5+ IL13+ and CD25+ FoxP3+ of CD4+ T cells were measured by flow cytometry from the homogenized lungs. The data are representative of two independent experiments and shown as the mean ± SEM (n = 8–15). (*) and (+) represent comparison of PBS vs OVA and Wt OVA vs KO OVA treatment groups respectively. *^/+^P<0.05, **^/++^P<0.01, ***^/+++^P<0.001.

We wanted to understand if the observed reduction of Th2 responses was OVA specific or rather a general trait of β5i KO mice. Thus, Wt and β5i KO mice were intranasally challenged with HDM (Figure 9A) followed by differential BAL cell counts and T-cells analysis. HDM application caused robust allergic inflammation as characterized by significant increase in BALs lymphocytes, eosinophils and neutrophils (Figure 9B). Similarly to OVA induced asthma, HDM challenge led to significantly elevated levels of lung Th2 and Treg cells (Figure 9C). However, no substantial difference in response to HDM was observed between Wt and β5i KO mice. These results ruled out that β5i KO mice have an inherited inability to mount an efficient Th2 response.

## Discussion

The present study focused on the role of β5i proteasome immunosubunit in experimental acute asthma. Currently, there is no information available on the role of this subunit in Th2 driven pathologies, while numerous reports have shown that β5i plays a role in Th1- and Th17- driven experimental inflammation such as DSS-induced colitis [14], [15], Crohńs disease and ulcerative colitis [12], [23] and collagen-induced arthritis [16]. Because non-specific proteasome inhibition by Bortezomib and PS-519 has been shown to significantly reduce airway eosinophilia [17], [18] in the models of acute asthma. Therefore, we hypothesized that β5i can also be involved in asthma development and first investigated its expression in healthy and asthmatic lungs of mice. Various methods including proteasome activity assays, two-dimensional gel electrophoresis and western blots confirmed strong expression of the β5i immunosubunit in the lung which however was not altered between naïve and asthmatic lungs (data not shown).

Induction of OVA/Alum asthma in β5i KO mice revealed impaired Th2 responses characterized by reduced eosinophilia, decreased numbers of pulmonary Th2 cells and OVA- specific IgG1 titers. In fact, this finding demonstrates for the first time the impact of the β5i immunosubunit on eosinophil recruitment directly into the lung. Notably, blood eosinophils of non-asthmatic β5i KO mice were comparable to normal control mice. In agreement with previous data from the colitis model [14], β5i KO mice also exhibited reduced amounts of neutrophils in the BAL during the asthma. Interestingly, the frequency of Th1 cells and Tregs in the lung of β5i KO mice was similar to Wt controls suggesting that they are not involved in the down regulation of the Th2 response. Histological analysis of goblet cell numbers and mucus secretion surprisingly revealed no differences between β5i mutant and Wt mice (data not shown).

Next, we wondered whether the reduced Th2 response is airway specific or rather a general trait of these mice. Thus, splenocytes of β5i KO and control mice were *in vitro* restimulated with OVA and production of IL-4, IL-5 and IL-13 was analyzed. Data revealed that cytokine secretion was reduced in β5i KO, indicating a systemic defect in OVA sensitization. There is substantial evidence that inhibition of the β5i proteasome subunit ameliorates autoimmune pathologies mainly via reduction of Th1 and Th17 cytokines and/or blocking the humoral antibody response [14], [15], [16]. Yet, the precise mechanisms and pathways by which β5i may affect these immunological processes is not completely understood. Possibly, the β5i subunit mediates its broad regulatory effects in the mucosal systems via activation of NF-κB in adaptive and innate immune cells.

Dendritic cells require NF-κB signaling for responding to exogenous stimuli and subsequent maturation [24], [25]. Further, the adjuvant effect of Alum is mediated via interaction with dendritic cells [20]. Therefore, we wanted to exclude any adjuvant mediated effect by employing the Alum independent, subcutaneous OVA asthma model. Similarly, to the OVA/Alum asthma, similar results were achieved with the alum-free protocol, demonstrating that Alum has no effect on reduced Th2 responses in β5i KO mice. While the OVA model of allergic inflammation shows several limitations with respect to the human disease [26], we were interested whether a similar phenotype also occurs in the clinically more relevant HDM asthma model. To our surprise, Th2 responses of HDM acute asthma were similar between Wt and β5i KO mice. To further substantiate this, we tested *in vitro* Th2 cell differentiation of naïve CD4+ T cells from β5i KO mice and found normal production of IL-4, IL-5 and IL-13 by these cells. We thus concluded that β5i KO mice are capable of mounting normal Th2 response to the different set of allergens, thus demonstrating that these mice do not show an inherited inability to mount normal Th2 responses which is in accordance with a recent publication by Kalim et. al. [27].

It has to be noted that there are major differences concerning the antigenicity between OVA and HDM. While OVA does not cause allergy without prior subcutaneous or intraperitoneal immunization [21], HDM is allergenic by itself. HDM is a complex mixture of *Dermatophagoides pteronyssinus* antigens which stimulates bronchial epithelial cells to produce eosinophil-attracting/Th2 cytokines such as GM-CSF, TSLP, IL-25 and IL-33, which in turn prime subsequent allergic inflammation [28]. In contrast to HDM, challenging mice with OVA without prior sensitization develops tolerance [29], [30]. Multiple sensitization steps with OVA/Alum induce formation of memory T and B-cells, which, after OVA aerosol challenges provoke asthma. We speculate that a possible failure to develop an efficient memory T and B-cells response towards OVA in β5i KO mice could partially explain the observed phenotype.

Immunoproteasome and β5i are constitutively expressed in lymphoid tissues and especially in immature dendritic cells, where they activate NF-κB and degrade byproducts of inflammation induced cellular oxidative stress [22], [31], [32]. It has been shown that intratracheal instillation of naïve mice with OVA pulsed BMDCs is sufficient to induce asthma after OVA aerosol challenge [33] and that depletion of lung CD11c+ dendritic cells during OVA challenge abrogates allergic inflammation [34]. In line with this, we assumed that β5i KO mice have defects in the inflammatory dendritic cells (iDCs) compartment. To this end, we investigated FITC-OVA uptake and maturation of lung iDCs from β5i KO mice under control and asthmatic conditions. Indeed, β5i KO iDCs showed reduced FITC-OVA uptake and maturation but normal migration into the mediastinal lymph nodes. Complementation of these defects by adoptive transfers of *in vitro* generated Wt BMDCs into β5i KO mice led to significantly increased allergic inflammation in Wt but not β5i KO mice, although the transfer equally increased iDCs, BAL neutrophils and Th1 cells in the lungs of both groups. The impaired antigen uptake and maturation of iDCs from β5i KO might thus be responsible for the low numbers of BAL neutrophils during OVA asthma but not for the decreased Th2 responses. However, neutrophil influx plays an important role in chronic allergic asthma and acute exacerbations of COPD [35], which makes β5i subunit a possible target for pharmacological intervention in these pathologies.

In summary, we show that the β5i immunosubunit is necessary for mounting a strong Th2 response in the OVA asthma model. Deficiency of this subunit results in a systemically impaired Th2 response, independent of β5i expression on DCs. The observed phenotype is OVA specific as no attenuation is detected in response to HDM allergens. β5i deficient naïve CD4+ T cells differentiate into normal IL-4, IL-5 and slightly elevated IL-13 producing Th2 cells, suggesting intact Th2 specific transcriptional program. Despite defects of lung iDCs from β5i KO mice in the uptake of antigen and maturation, they are not responsible for the reduced Th2 phenotype during Ova asthma. Further molecular and immunological studies have to be performed in order to fully elucidate the mechanisms of the observed β5i phenotype.

## Materials and Methods

### Ethics statement

All experiments with animals were performed corresponding to international and German guidelines on animal research and ethics. The used protocols were approved by Regierungspräsidium Gießen, Hessen, Germany. Permit number: MR 20/6 Nr.71/2011.

### Animals

Female and male 8–10 weeks old C57BL/6 mice were purchased from Charles River Laboratories, Germany. Female and male β5i KO mice on C57BL/6N background were obtained from the Max Planck Institute for Infection Biology (Berlin, Germany). Both genders were used in the same ratio in all the experiments.

### Mouse models of acute allergic airway inflammation

The OVA/Alum-induced allergic airway inflammation model was induced as described previously [21], except that mice were sensitized at days 0, 14 and 21. Control mice were sensitized with 1.5 mg Al(OH)_3_ (Pierce, Rockford, IL, USA) in 200 µL PBS. Challenges with 1% OVA aerosol were performed at days 26, 27 and 28. For investigation of lung inflammatory dendritic cells antigen uptake and maturation mice were challenged daily from day 26 until day 32. On day 32, 100 µg FITC-labeled OVA was applied i.n. (OVA was labeled by FluoReporter FITC Protein Labeling Kit, Life Technology, Darmstadt, Germany; performed as described in the manufacturer's protocol, degree of labeling was 7 FITC-molecules per OVA-molecule). For BMDCs transfer experiment mice were challenged daily from day 26 until day 32 and BMDCs were injected i.p. at days 14, 21, 28 and 30. The adjuvant free, subcutaneous allergic airway inflammation model was performed as described previously [21]. House dust mite (HDM) allergic asthma was induced by weekly i.n. challenges of slightly anesthetized mice (i.p. injection of Ketamine/Rompun (Ketamin-Inresa; Inresa Arzneimittel GmbH, Freiburg, Germany)/Rompun 2% (Bayer, Leverkusen, Germany) with 100 µg HDM extract (GREER, Lenoir, USA) in 50 µL PBS on days 0, 7, 14 and 21. One day after last OVA or two days after last HDM challenges mice were sacrificed and blood, bronchoalveolar lavage fluids (BAL), lungs and spleens were collected for further analysis.

### Bronchoalveolar lavage

BAL was performed by using 1 mL ice-cold PBS containing 1× protease inhibitor cocktail (Roche, Manheim, Germany). BAL cells analysis was performed as previously described [21]. BAL fluids were centrifuged and the supernatants were stored at -20 °C until cytokine were measured. The measurement of cytokines IL-4, IL-5 and IL-13 was performed by Cytometric Bead Array (BD, Heidelberg, Germany) according to manufacturer's protocol. Sensitivities for measured cytokines were 0,3 pg/mL for IL-4, 0,9 pg/mL for IL-5 and 2,4 pg/mL for IL-13.

### Measurement of OVA-specific antibodies levels

OVA-specific IgG1 antibody titers were measured in the serum as described previously [21].

### Spleen cells restimulation and cytokine analysis

Spleens were harvested and single cell suspensions were prepared. Erythrocytes were lysed and the cell concentration was adjusted to 2×10^6^ cells/mL by RPMI-1640 supplemented with 10% FCS, 5 mM glutamine, 100 µg/ml streptomycin, 100 U/ml penicillin. Next, 200 µL of the suspension were transferred into a 24-well plate and restimulated with 50 µg/mL OVA (grade VI) for 72 h (37°C, 5% CO_2_). Then plates were centrifuged (350 g, 10 min), supernatants were collected and stored at −20°C until the cytokines measurement. IL-4, IL-5 and IL-13 cytokines quantification was performed by Cytometric Bead Array (BD, Heidelberg, Germany) according to the manufacturer's protocol.

### Flow cytometry

Lungs were cut into 2–5 mm pieces and incubated for 30 min at 37°C in the incubation medium (IM) (RPMI-1640 with L-Glutamine and NaHCO_3_, 10% FCS, 1× non-essential amino acids (PAA, Pasching, Austria), 100 µg/mL streptomycin, 120 µg/mL penicillin) supplemented with1 mg/mL Collagenase D (Roche, Basel, Switzerland) and 20 µg/mL DNase I (Roche, Basel, Switzerland). The predigested lungs were minced through 100 µm nylon cell strainer (BD Falcon, NJ, USA) diluted with IM and centrifuged at 1700 rpm for 5 min. Cell pellets were resuspended in erythrocytes lysis buffer (8 g/L NH_4_Cl; 1 g/L KHCO_3_; 37,2 mg/L EDTA) and incubated at room temperature for 3–5 mins. Then, cells were washed with IM, centrifuged and resuspended in IM supplemented with 50 ng/mLPMA, 750 ng/mL ionomycin and 10 mg/mL brefeldin A for 4 h. DCs and T-cells extracellular staining was performed in PBS with 1% FCS and presence of BD Fc Block anti-CD16/CD32 (93) (BD, Heidelberg, Germany) and fluorophore-labeled antibodies (purchased from eBioscience, San Diego, USA, if not specified otherwise). The following antibodies for DCs extracellular staining were used: anti-F4/80-APC (C1:A3-1); anti-CD11b-eFluor 450 (M1/70); anti-CD11c-APC-eFluor780 (BU15); anti-CD45R/B220-V500 (RA3-6B), (BD, Heidelberg, Germany), anti-Ly-6C-AlexaFluor 700 (HK1.4) and anti-I-A/I-E-PerCP-Cy5.5 (M5/115.15.2) from Biolegend, San Diego, USA. Extracellular T-cell staining was performed with anti-CD4-V450 (RM4-5) anti-CD8a-V500 (53-6.7) and anti-CD25-PE-Cy7 (PC61.5) (BD, Heidelberg, Germany); After extracellular staining, T-cells were fixed with FoxP3-Fixation Kit (eBioscience, San Diego, USA) and permeabilized with 0,3% Saponin, 1% FCS in PBS followed by an intracellular staining in presence of BD Fc Block anti-CD16/CD32 (93) (BD, Heidelberg, Germany) with the following fluorophore-labeled antibodies: IFNγ-PerCP-Cy5.5 (XMG1.2) (Biolegend, San Diego, USA) and FoxP3-AlexaFluor 700 (FJK-16 s); IL-5-PE (TRFK5); IL-13-AlexaFluor 647 (eBio13A), (eBioscience, San Diego, USA). Every staining included respective negative and isotype controls. Fluorescence signals were acquired by flow cytometry (FACSAria III;BD, Heidelberg, Germany) and analyzed using FACSDiva™ software.

### Bone marrow derived dendritic cells (BMDCs) preparation

BMDCs preparation and growth was performed as described in [36] with modifications. Bone marrow was isolated from femur and tibia and a single cell suspension was prepared in dendritic cell incubation medium (DCIM; RPMI-1640 (with Hepes; PAA, Pasching, Austria) supplemented with 10% FBS (Life Technologies GmbH, Darmstadt, Germany), 0,5% β-Mercaptoethanol, 1% Penicillin, 1% Streptomycin and 1% L-Glutamin (Life Technologies GmbH, Darmstadt, Germany)). Cell pellets were resuspended in erythrocytes lysis buffer (8 g/l NH_4_Cl; 1 g/l KHCO_3_; 37,2 mg/l EDTA) and incubated at room temperature for 3–5 mins. Then, cells were washed, centrifuged and resuspended in DCIM. About 4×10^6^ cells were plated in Nunclon™Δ Surface Petri dishes (Nalge Nunc International, Rochester, USA) in 10 mL DCIM supplemented with 10% GM-CSF (supernatant of murine hybridoma cell line ×6310). Freshly prepared DCIM was added to the cell suspension at days 3 (supplemented with 10% GM-CSF) and 6 (supplemented with 10% GM-CSF and 300 µg/ml OVA). As control, one batch of DCs was prepared without any OVA stimulation. On day 7 cells were washed twice with PBS, and injected i.p. in PBS at amount of 0.8×10^6^ cells per mouse.

### T-cells *in vitro* differentiation

Naive CD4^+^ CD62L^hi^ cells were isolated from spleens and lymph nodes of WT and β5i KO mice by magnetic associated cell sorting (MACS, Miltenyi Biotec, Bergisch-Gladbach, Germany). Isolated cells were differentiated into Th2 cells in the presence of plate-bound anti-CD3 (5 µg/ml, clone 145-2C11), soluble anti-CD28 (1 µg/ml, clone 37.51), anti-IFN-γ antibodies (XMG1.2) 5 µg/ml and recombinant murine IL-4 20 ng/mL (PeproTech, Rocky Hill, USA). After 3 days of incubation, cells were transferred into new plates containing IL-2 100 U/ml and incubated further for the next 3 days. On the last incubation day cells were restimulated and analyzed by Flow Cytometry as described above.

### Statistical analysis

Data were expressed as mean ± standard error of mean (SEM). One-way ANOVA statistical analysis was performed followed by Tukey's HSD test using GraphPad Prism 5 Software. P-values of P<0.05 were considered significant.

## Supporting Information

Figure S1 **Representative lung Th2 cells staining of Wt and β5i KO mice during OVA/Alum acute asthma.** The percentages of IL5+ IL13+ of CD4+ T cells were measured by flow cytometry from the homogenized lungs. The presented plots correspond to the data points of Th2 cells in Figure 2. The gating strategy was determined by comparing the stained samples to the negative and isotype controls.
(TIF)

Figure S2 **Representative lung Treg cells staining of Wt and β5i KO mice during OVA/Alum acute asthma.** The percentages of CD25+ FoxP3+ of CD4+ T cells were measured by flow cytometry from the homogenized lungs. The presented plots correspond to the data points of Treg cells in Figure 2. The gating strategy was determined by comparing the stained samples to the negative and isotype controls.
(TIF)

Figure S3 **Gating strategy for identification of lung inflammatory dendritic cells (iDCs).** Macrophages were excluded based on the F4/80 staining. F4/80 negative cells were further gated to exclude CD11c negative cells. CD11c positive cells were gated to exclude CD11b negative cells. Double positive CD11c+ CD11b+ population was separated into two groups based on the expression of Ly6C inflammatory monocyte precursor marker. In order to eliminate possible B-cells contamination triple positive CD11c+ CD11b+ Ly6C+ population was further gated to remove B220 positive cells. CD11c+ CD11b+ Ly6C+ B220−iDCs were analyzed on the presence of the uptaken OVA-FITC with the subsequent MHC II surface expression. The presented strategy corresponds to the data points of iDCs in Figure 6 and Figure S4A. The gating strategy was determined by comparing the stained samples to the negative and isotype controls.
(TIF)

Figure S4 **Increase in lung iDCs and Th1 cells upon BMDCs transfer to β5i KO mice during OVA/Alum acute asthma.** The numbers of iDCs and percentages of IFN−γ+ of CD4+ T cells were measured by flow cytometry from the homogenized lungs. The data are representative of two independent experiments and shown as the mean ± SEM (n = 6–12). (*) and (+) represent comparison between PBS vs OVA and Wt OVA vs KO OVA treatment groups respectively. (#) represents comparison of Wt OVA vs Wt DCs OVA. *^/+/#^P<0.05, **^/++^P<0.01, ***^/+++^P<0.001.
(TIF)

Figure S5 **Representative staining of** ***in vitro*** **differentiated Th2 cells from naïve Wt and β5i KO CD4+ T cells.** The percentages of IL−4+, IL−5+ and IL−13+ of CD4+ T cells were measured by flow cytometry from *in vitro* differentiated Th2 cells. The presented plots correspond to the data points in Figure 9. The gating strategy was determined by comparing the stained samples to the negative and isotype controls.
(TIF)
