# Supplementary figures and images for: β5i Subunit Deficiency of the Immunoproteasome Leads to Reduced Th2 Response in OVA Induced Acute Asthma
Source: PLoS One. 2013 Apr 4;8(4):e60565. doi: 10.1371/journal.pone.0060565 (PMC3617144; doi:10.1371/journal.pone.0060565)

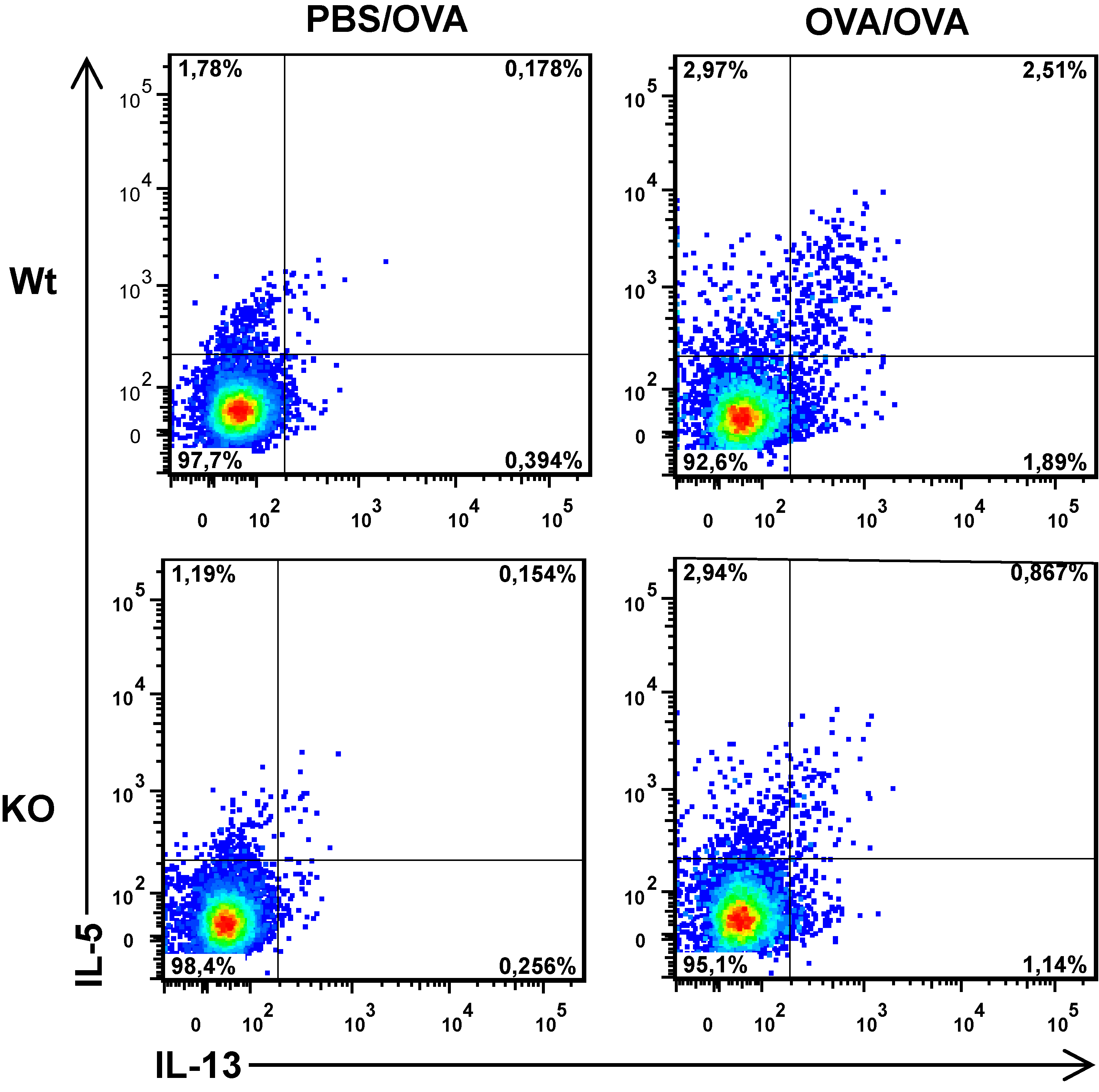

Supplement: Figure S1 — Representative lung Th2 cells staining of Wt and β5i KO mice during OVA/Alum acute asthma. The percentages of IL5+ IL13+ of CD4+ T cells were measured by flow cytometry from the homogenized lungs. The presented plots correspond to the data points of Th2 cells in Figure 2. The gating strategy was determined by comparing the stained samples to the negative and isotype controls. (TIF) [file pone.0060565.s001.tif]

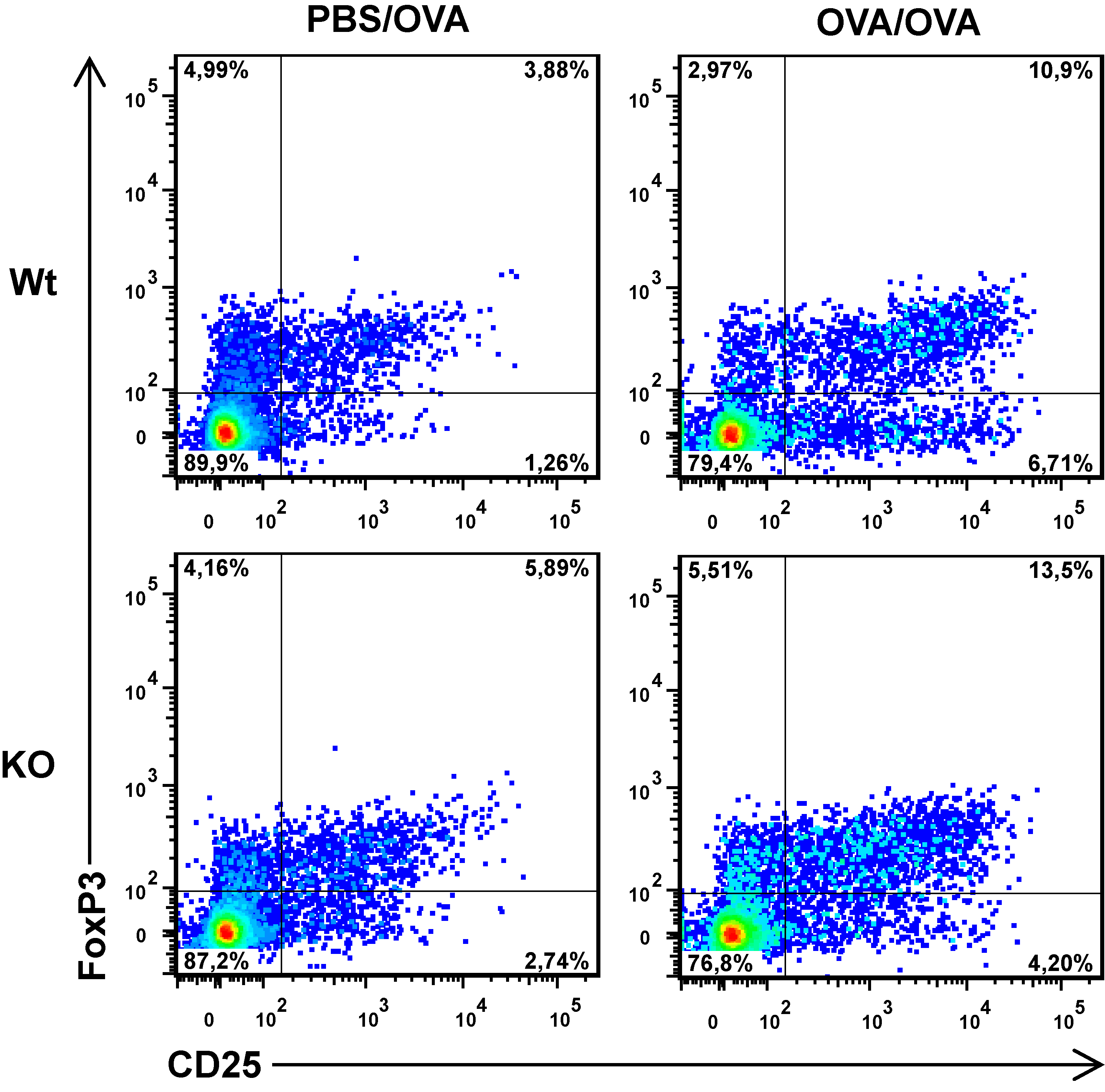

Supplement: Figure S2 — Representative lung Treg cells staining of Wt and β5i KO mice during OVA/Alum acute asthma. The percentages of CD25+ FoxP3+ of CD4+ T cells were measured by flow cytometry from the homogenized lungs. The presented plots correspond to the data points of Treg cells in Figure 2. The gating strategy was determined by comparing the stained samples to the negative and isotype controls. (TIF) [file pone.0060565.s002.tif]

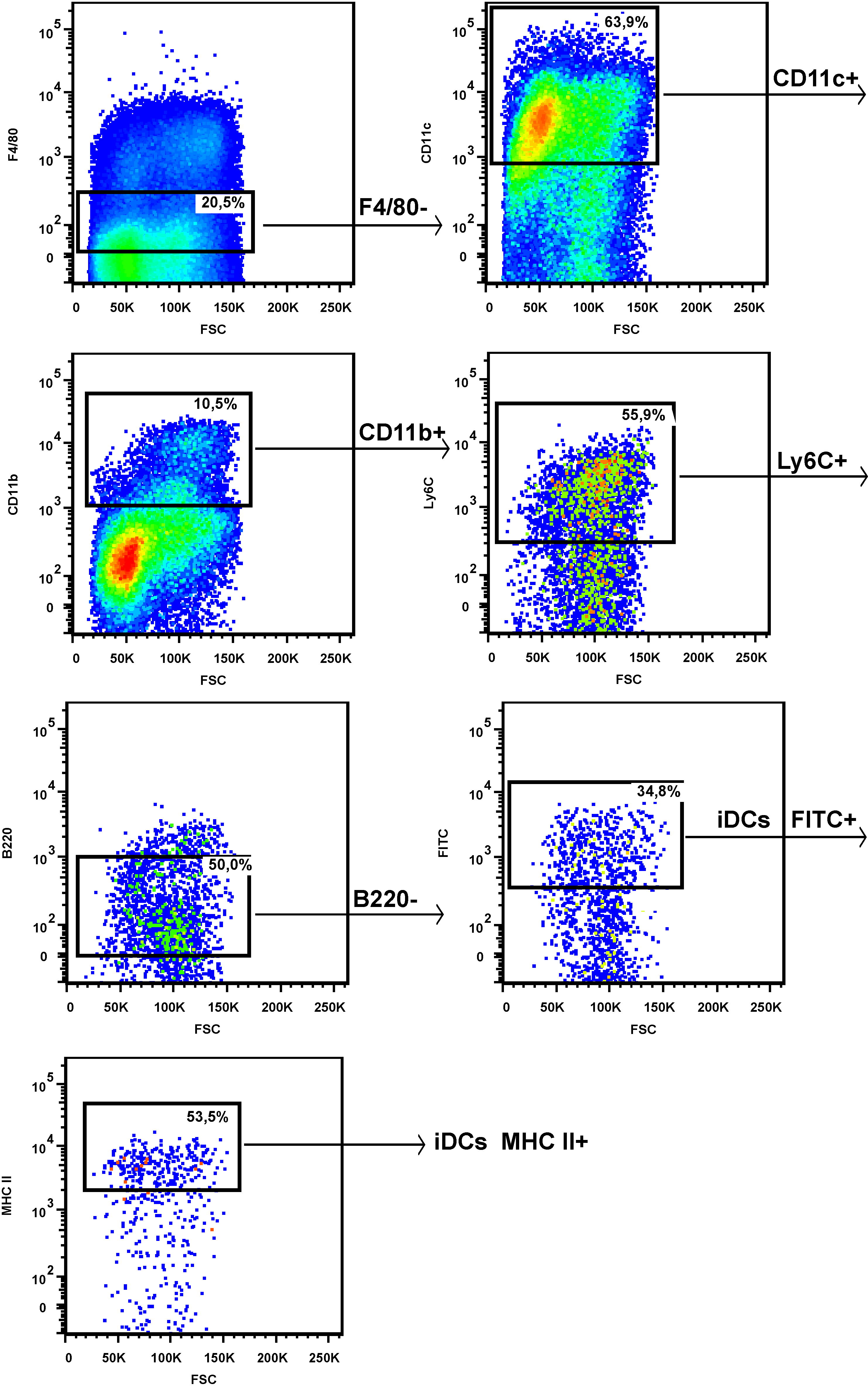

Supplement: Figure S3 — Gating strategy for identification of lung inflammatory dendritic cells (iDCs). Macrophages were excluded based on the F4/80 staining. F4/80 negative cells were further gated to exclude CD11c negative cells. CD11c positive cells were gated to exclude CD11b negative cells. Double positive CD11c+ CD11b+ population was separated into two groups based on the expression of Ly6C inflammatory monocyte precursor marker. In order to eliminate possible B-cells contamination triple positive CD11c+ CD11b+ Ly6C+ population was further gated to remove B220 positive cells. CD11c+ CD11b+ Ly6C+ B220−iDCs were analyzed on the presence of the uptaken OVA-FITC with the subsequent MHC II surface expression. The presented strategy corresponds to the data points of iDCs in Figure 6 and Figure S4A. The gating strategy was determined by comparing the stained samples to the negative and isotype controls. (TIF) [file pone.0060565.s003.tif]

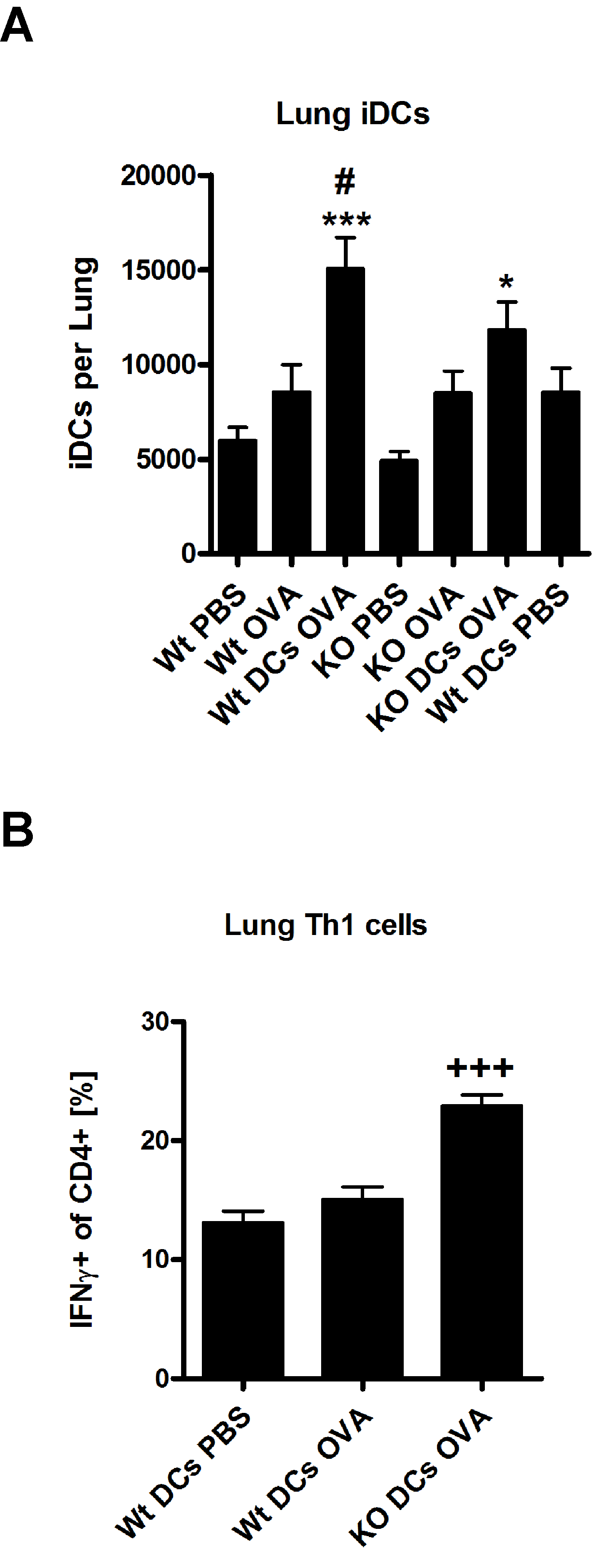

Supplement: Figure S4 — Increase in lung iDCs and Th1 cells upon BMDCs transfer to β5i KO mice during OVA/Alum acute asthma. The numbers of iDCs and percentages of IFN−γ+ of CD4+ T cells were measured by flow cytometry from the homogenized lungs. The data are representative of two independent experiments and shown as the mean ± SEM (n = 6–12). (*) and (+) represent comparison between PBS vs OVA and Wt OVA vs KO OVA treatment groups respectively. (#) represents comparison of Wt OVA vs Wt DCs OVA. */+/#P<0.05, **/++P<0.01, ***/+++P<0.001. (TIF) [file pone.0060565.s004.tif]

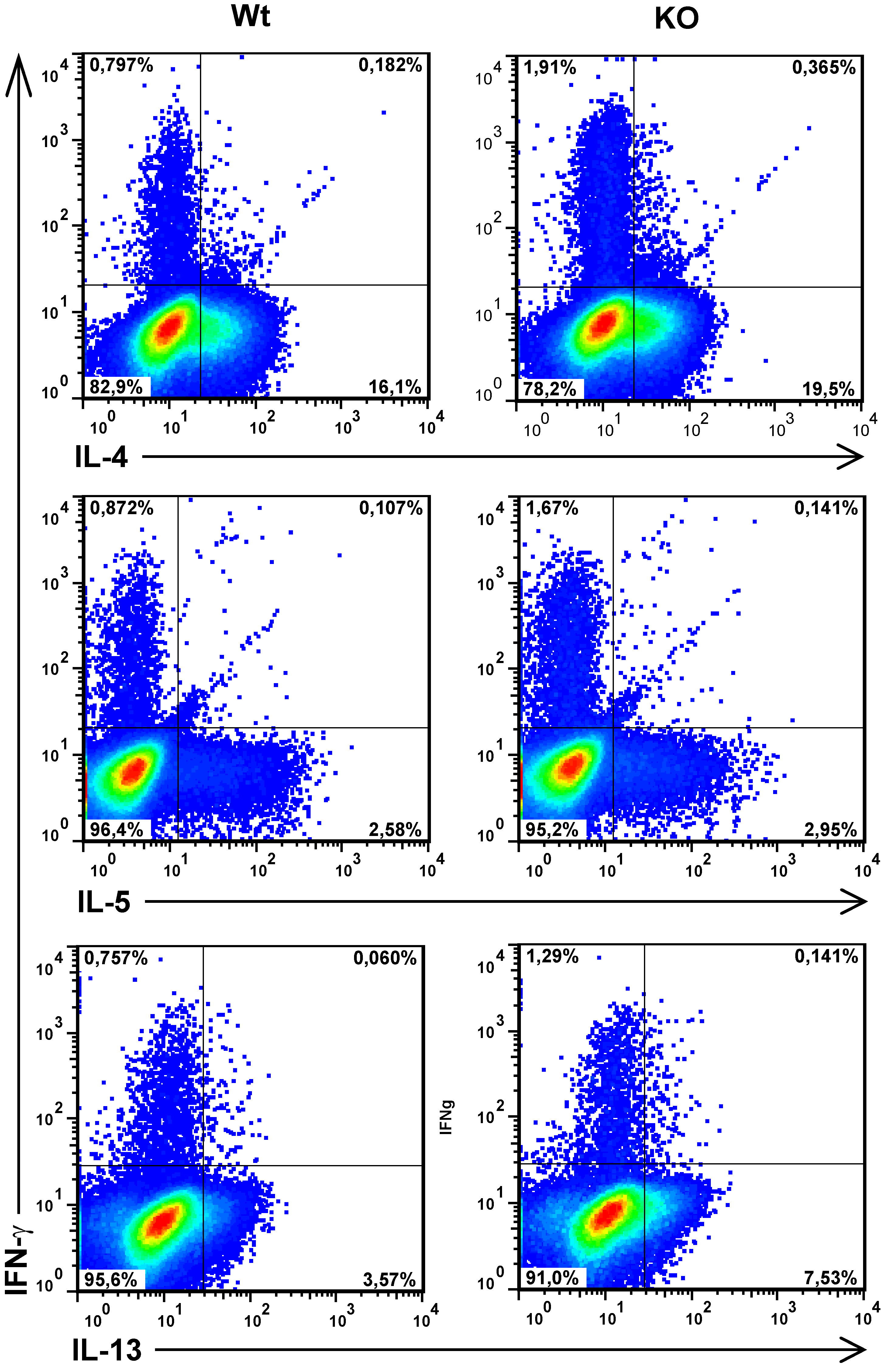

Supplement: Figure S5 — Representative staining of in vitro differentiated Th2 cells from naïve Wt and β5i KO CD4+ T cells. The percentages of IL−4+, IL−5+ and IL−13+ of CD4+ T cells were measured by flow cytometry from in vitro differentiated Th2 cells. The presented plots correspond to the data points in Figure 9. The gating strategy was determined by comparing the stained samples to the negative and isotype controls. (TIF) [file pone.0060565.s005.tif]
